# Poor adherence to the World Health Organization guidelines of treatment of severe pneumonia in children at Khartoum, Sudan

**DOI:** 10.1186/1756-0500-7-531

**Published:** 2014-08-14

**Authors:** Karim Eldin M Salih, Jalal A Bilal, Mona A Alfadeel, Yassin Hamid, Widad Eldouch, Elfatih Elsammani, Salah A Ibrahim, Ishag Adam

**Affiliations:** Department of Pediatrics, College of Medicine, King Khalid University, Abha, Saudi Arabia; College of Medicine, Qassim University, Buraydah, Saudi Arabia; King Khalid University Hospital, Riyadh, Saudi Arabia; Almaarifa College for Science and Technology, Khartoum Children, Riyadh, Saudi Arabia; Department of epidemiology, Federal Ministry of Health, Khartoum, Sudan; Department of Epidemiology, School of Medicine, Ahfad University for Women, Omdurman, Sudan; Faculty of Medicine, University of Khartoum, Khartoum, Sudan

**Keywords:** Pneumonia, Treatment, Adherence, Penicillin, Sudan

## Abstract

**Background:**

Community-acquired pneumonia (CAP) is as a major cause for childhood morbidity and mortality worldwide. This study was conducted to investigate the adherence and response of the WHO guidelines for treatment of severe pneumonia.

**Method:**

A cross-sectional study was conducted in the period of June 2009 to July 2010 at Khartoum Hospital, Sudan. Children admitted and treated for severe pneumonia were enrolled.

**Results:**

Only 39 (18.8%) out of 208 enrolled children received prescriptions that were adherent to the WHO guidelines of treatment of severe pneumonia. In logistic regression none of the investigated variable (age, gender, and clinical presentations) was associated with the adherence to the WHO guidelines. There was no significant difference in the response between adherent and non-adherent prescriptions. There was no association between the demographic, clinical data, treatment-adherence to the guidelines and the patients’ response.

**Conclusion:**

There is a poor (18.8%) adherence to the WHO guidelines of the treatment of severe pneumonia in the region regardless to the age, gender and clinical presentation.

## Introduction

Community-acquired pneumonia (CAP) is as a major cause for childhood morbidity and mortality worldwide [1]. World Health Organization has estimated an annual global incidence of new episodes of pneumonia as156 million, the vast majority of these (151 million) episodes were reported from the developing countries [2]. The leading bacterial cause of pneumonia in underdeveloped countries is pneumococcus (30–50%), *H. influenzae* type b (10–30% of cases), *S. aureus* and *K. pneumonia*e [3]. Empiric antibacterial selection for treatment of childhood CAP must be based on age and the likely causative organisms [4]. Early and prompt diagnosis of bacterial pneumonia is lacking in most of the developing countries, hence empirical treatment is usually practiced. Sudan has one of the highest infant mortality rates (112/1000 live birth) and respiratory infections are among the most killing causes [5]. There is no published data addressing pneumonia/treatment in Sudan. Most hospital guidelines in developing countries recommend the use of penicillin and its derivatives for treatment of severe pneumonia based on the WHO guidelines [6]. Generally, many clinicians are concerned that guidelines do not reflect the complexity of the real situation and may stand against those who advocate its application in clinical practice [7].

The objectives of this study were to determine the adherence to the WHO guidelines for treatment of severe pneumonia and to compare the response to the treatment between the adherences and an empirical antibiotic regimen initially used in the treatment of patients with severe CAP at the end of 48 hours of starting antibiotic in a major pediatric facility in Sudan.

## Methods

A longitudinal hospital-based study was conducted at the Children Emergency Hospital (CEH), Khartoum, Sudan in the period from June 2009 to July 2010. CEH is the main urban children referral hospital in Sudan with the capacity of 240 beds and a catchment population of more than five million people representing the population of the tripartite metropolis, Khartoum [8].

After signing an informed consent (by the parents/guardian) all consecutively admitted children aged 2 to 59 month with definite labeling diagnosis of “severe pneumonia”, based on the WHO criteria for diagnosis of pneumonia were included [6]. The critically ill children, children with known TB, HIV, bronchial asthma, other chronic conditions that might be complicated by pneumonia and those who refuse to be enrolled in the study were excluded. A radiologist reviewed the chest radiographs (CXR) taken for every child satisfying the WHO criteria for severe pneumonia at the time of admission. Only those with evidence of lobar or segmental consolidation or with an infiltrate in at least one lung on chest radiographs were included.

The investigators recorded demographic, clinical and treatment data as prescribed and written by the treating physicians from patients files at the time of admission and at day 2 to determine response or failure to the antibacterial treatment. Evaluation data include symptoms (cough, inability to drink, vomiting, lethargy), clinical signs (tachypnea, temperature of ≥37.5°C, signs of respiratory distress, grunting and full chest examination). A patient was labeled “responding” if the respiratory rate returned to age specific normal range, less fever and eating better after 48 hours following prescription [6]. The patient was labeled “not responding” if respiratory rate did not return to age-specific normal at the end of 48 hours of antibiotic treatment, still febrile and/not eating better.

### Ethical consideration

The Development and Ethics Committee of Khartoum Children Emergency Hospital approved this study prior to collecting data.

### Statistical analyses

Data were entered and analyzed into SPSS statistical software version 16.0 (SPSS Inc., Chicago, IL, U.S.A. 2007). The descriptive analyses used included the mean, standard deviation, and frequency distribution. Chi square test was used to compare the difference between categorical data. Univariate and multivariate analyses were performed where adherence (penicillin prescription) was the dependent variable and the other variables (age, gender, symptoms) were the independent variable. Another model was performed for the outcome of treatment. Odds ration and 95% confidence interval (CI) were calculated and P < 0.05 was considered significant.

## Results

Two hundred and eight children fulfilled the inclusion criteria in this study. Table 1 shows the demographic and clinical characteristics of the studied group. The mean (SD) of the age was 28.12 (13.9) months and 99 (47.6%) were males. Only 39 (18.8%) of these children received the prescription that was adherent to the WHO guidelines (penicillin) of severe pneumonia.

**Table 1:** **Clinical manifestation of the children**

Variable	Frequency (%)
Fever	196 (94.2)
Active alae nasi	175 (93.1)
Intercostal recession	174 (92.6)
Shortness of breathing	192 (92.3)
Cough	181 (88.3)
Runny nose	158 (85.4)
Chest indrawing	154 (81.9)
Grunting	135 (73.4)

The antimicrobial prescriptions, which were not in accordance with the WHO guidelines, were: amoxicillin/clavulanic acid (46, 22.1%), ceftriaxone (42, 20.2%), cefuroxime (41, 19.7%), a combination of penicillin/gentamicin (29; 13.9%) and others 11 (11, 5.3%).

None of the investigated factors (age, gender, symptoms and signs) were found as predictors for adherence of the WHO guidelines for treatment of severe pneumonia, Table 2.

**Table 2:** **Factors associated with adherence to WHO guidelines of treatment of severe pneumonia in the study using univariate analyses**

Variables	OR (95% CI)	P value
Age	0.9 (0.9 − 1.1)	0.902
Sex	1.3 (0.6 − 2.81)	0.380
Fever	0.8 (0.1 − 4.0)	0.849
Cough	0.8 (0.2 − 26.9)	0.530
Runny nose	0.9 (0.4 − 2.2)	0.913
Breathlessness	0.5 (0.1 − 2.7)	0.742
Grunting	0.8 (0.3 − 1.9)	0.838
Active alae nasi	1.1 (0.3 − 3.7)	0.761
Intercostal recession	1.8 (0.5 − 6.1)	0.304
Chest indrawing	1.1 (0.4 − 2.8)	0.645

There was no significant difference in the response between adherence and non-adherence prescriptions [32/39 (82.1%) vs. 151/169 (81.2%), P = 0.206]. None of the investigated predictors was found to be associated with treatment outcome as shown, Table 3.

**Table 3:** **Factors associated with outcome of treatment of severe pneumonia in children in the study using univariate and multivariate analyses**

Variables	Univariate		Multivariate	
	OR (95% CI)	P	OR (95% CI)	P
Adherence	0.5 (0.2 − 1.4)	0.271	2.3 (0.8 − 6.6)	0.099
Age	0.8 (0.18 − 4.09)	0.849	0.9 (0.9 − 1.1)	0.255
Sex	0.8 (0.2 − 2.6)	0.530	0.8 (0.3 − 2.1)	0.701
Clinical	0.9 (0.4 − 2.2)	0.913	1.0 (0.3 − 3.4)	0.925
Cough	0.9 (0.2 − 3.5)	0.961	1.2 (0.2 − 5.8)	0.753
Runny nose	2.9 (1.2 − 7.1)	0.017	0.3 (0.1 − 1.4)	0.065
Breathlessness	2.1 (0.2 − 16.9)	0.470	0.4 (0.1 − 4.0)	0.479
Grunting	0.6 (0.2 − 1.3)	0.429	1.6 (0.5 − 4.9)	0.376
Active alae nasi	1.1 (0.2 − 5.4)	0.834	0.7 (0.9 − 5.7)	0.770
Intercostal recession	1.8 (0.2 − 14.6)	0.567	0.3 (0.1 − 2.9)	0.362
Chest indrawing	2.0 (0.2 − 15.9)	0.512	2.2 (0.5 − 4.9)	0.116

## Discussion

The WHO has stressed on the accountability and accessibility of the health information and data at the country level to measure and monitor results. The data collection on children health is essential to determine for focusing of investments and progress monitoring [9]. The main findings in this study were that there was poor adherence to the WHO guidelines of treatment of severe pneumonia in under-five year old children. The almost 82% of non-adherence to the WHO guidelines of severe pneumonia in children indicated that patients did not receive the appropriate medication to their clinical needs and the antibiotics were not at the lowest cost to them and their community. This almost defines an irrational use of antibiotics [10]. The Sudan Association of Paediatricians developed a local WHO-based protocol for treatment of emergency conditions in children including pneumonia in 2011. Though this protocol was not validated and did not take effect in clinical practice, yet the adherence in this study was not in compliance with its treatment guidelines [11]. The non-adherence rate was higher than that reported from Burkina Faso. Their estimate was around 61% though it was an estimate of compliance for pneumonia among other diseases yet it is here as well [12]. Moreover the prescribing personnel, in their report, were nurses and health workers and not physicians as in our study. In a Tanzanian study, half of the children with severe pneumonia were prescribed antibiotics, which are not recommended by the WHO [13]. Even in developed countries, the compliance to privileged countries guidelines is not usually perfect despite good training, a fact, which was illustrated in one study where even, restricted initial broad-spectrum antimicrobial therapy to cultured pathogens was a rare practice [14]. The statement of the guidelines might not be clear, especially for junior physicians as well as it was not endorsed by the senior physicians who used to train them. Tis might, possibly be, because they were not convinced of the recommended antibiotic in the guidelines however, studies on knowledge attitude and behavior towards formal guidelines may well explain this point.

The fact that there was no difference in treatment outcome when following the WHO guidelines or using alternative antibiotics, in this study, may serve adherence to the guidelines until prove otherwise. A similar finding was reported in a Kenyan survey where poor guideline adherence was attributed to preferences for broader spectrum, non-beta-lactam antibiotics [15]. Moreover, another Kenyan study reported a lower rate (48.3%) among children with pneumonia in which prescription had improved to 90% adherence after intervention (training and booklet distribution) [16]. This praises training as an important tool in improving adherence and thus better outcome of pneumonia in children.

Antibiotics prescribed in this study except for penicillin do not follow any of the international guidelines. Commonly used antibiotics for severe pneumonia in an African setting were penicillin, sulphonamides, and aminoglycosides [17]. Amoxicillin and sulphonamides were the commonly encountered prescriptions in management of pneumonia in other areas too e.g. Tanzania and rural Peru [12, 18]. The results of a Sudanese survey in prescribing patterns proved that at least some parts of population are unable to afford treatment for some important and common diseases including childhood pneumonia and an average 66% rate of antibiotics prescription [19]. The variable antibiotic prescription may depend not only on guidelines unacceptability among physicians but also on health provider characteristics, experience and attitude [15]. Moreover, the health facilities settings and equipment may be an important factor. This study showed no correlation between clinical presentation and treatment outcome of severe pneumonia in children. However, duration of fever, rather than mere fever and tachypnea were documented as strong predictors for diagnosis of pneumonia rather than outcome in one study [20]. Another study reported tachypnea and chest indrawing as best predictors of pneumonia severity [21]. An extended evaluation in further studies is amenable to assess the morbidity and mortality of guideline-based treatment pneumonia in this age group.

This study did not address the health system, physician and family factors that may influence the implementation of the guidelines and the long-term outcome of children such as mortality. It was not the aim of this work, albeit useful, to study the cure rate, the full recovery and mortality of the children with severe pneumonia. Moreover, prior antibiotic treatment before admission was not recorded as well as supportive therapy. However this study, to the best of our knowledge, is the first of its type to determine physicians’ adherence to health policies in Sudan. The relatively reasonable sample in this study enabled us to draw conclusions on the adherence to the widely practiced WHO guidelines and the pattern of antibiotic prescription in a developing country.

## Conclusion

The non-adherence to WHO guidelines of severe pneumonia is the highest in the region (82%) regardless to the age and gender. There was no difference in the response between adherence and non-adherence. Studies are needed to determine physician and family factors influencing the implementation of the guidelines and their effects on the long term.
